# Contrast-enhanced photon-counting detector CT for discriminating local recurrence from postoperative changes after resection of pancreatic ductal adenocarcinoma

**DOI:** 10.1186/s41747-025-00567-0

**Published:** 2025-02-22

**Authors:** Zlatan Alagic, Carlos Valls Duran, Anders Svensson-Marcial, Seppo K. Koskinen

**Affiliations:** 1 Department of Diagnostic Radiology, Karolinska University Hospital, 171 76 Stockholm, Sweden; 2 Department of Clinical Science, Intervention, and Technology (CLINTEC), Karolinska Institutet, 171 77 Stockholm, Sweden

**Keywords:** Carcinoma (pancreatic ductal), Logistic models, Neoplasm recurrence (local), Sensitivity and specificity, Tomography (x-ray computed)

## Abstract

**Background:**

We evaluated the diagnostic capability of photon-counting detector computed tomography (PCD-CT) spectral variables in late arterial phase (LAP) and portal venous phase (PVP) to discriminate between local tumor recurrence (LTR) and postoperative changes (POC) after pancreatic ductal adenocarcinoma (PDAC) resection.

**Methods:**

Seventy-three consecutive PCD-CT scans in 73 patients with postoperative soft-tissue lesions (PSLs) were included, 42 with POC and 31 with LTR. Regions of interest were drawn in each PSL, and spectral variables were calculated: iodine concentration (IC), normalized IC (NIC), fat fraction, attenuation at 40, 70, and 90 keV, and slope of the spectral curve between 40–90 keV. Multivariable binary logistic regression models were constructed. Diagnostic performance was assessed for LAP and PVP using receiver operating characteristic analysis.

**Results:**

In LAP, all variables except fat fraction showed significant differences between LTR and POC (*p* ≤ 0.025). In PVP, all variables except NIC and fat fraction demonstrated significant differences between LTR and POC (*p* ≤ 0.005). Logistic regression analysis included NIC and 70 keV in the LAP-based model and IC and 90 keV in the PVP-based model. Both models achieved a higher area under the curve (AUC) than individual spectral variables in each phase. The LAP-based model achieved an AUC of 0.919 with 94% sensitivity, 84% specificity, and 87% accuracy, while the PVP-based model reached 0.820, 71%, 88%, and 81%, respectively.

**Conclusion:**

Spectral variables from PCD-CT help distinguish between LTR and POC in LAP and PVP post-PDAC resection. Multivariable logistic regression improves diagnostic performance, especially in LAP.

**Relevance statement:**

Measuring normalized iodine concentration and attenuation at 70 keV in late arterial phase, or iodine concentration and attenuation at 90 keV in portal venous phase, and incorporating these values into a logistic regression model can help differentiate between local tumor recurrence and postoperative changes after pancreatic ductal adenocarcinoma resection.

**Key Points:**

- Distinguishing recurrence from postoperative changes on CT after pancreatic ductal adenocarcinoma resection is challenging.
- PCD-CT spectral variable values differed significantly between local tumor recurrence (LTR) and postoperative changes (POC).
- Logistic regression of spectral variables can help distinguish LTR from POC.
- The late arterial phase-based model reached an AUC of 0.919 with 94% sensitivity and 84% specificity.

**Graphical Abstract:**

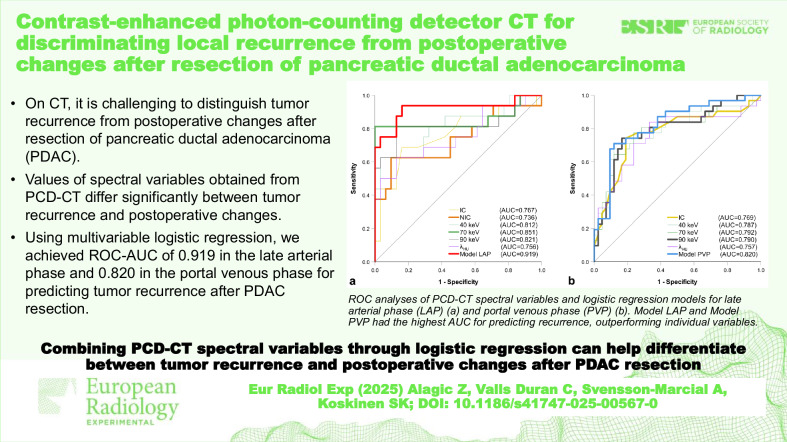

## Background

Pancreatic ductal adenocarcinoma (PDAC) carries a dismal prognosis attributable to its locally aggressive growth and early systemic spread [1]. Most patients present with advanced disease, with radical surgical resection feasible for only about 30% [2]. The proportion of patients eligible for surgical resection has risen following the introduction of neoadjuvant therapy for locally advanced tumors. In particular, neoadjuvant therapy with the chemotherapy protocol Folfirinox has achieved resectability rates of 60% in patients with initially unresectable tumors [3]. As neoadjuvant treatments have advanced, so too have surgical resection techniques, which continue to serve as the cornerstone in any curative treatment strategy for PDAC [4].

Isolated local recurrence occurs in up to 30% of the patients, and the treatment options are chemoradiotherapy, stereotactic body radiation therapy, and re-resection [5]. It has been shown that median survival after resection of isolated tumor recurrence was significantly longer compared to exploration without resection: 26.0 months *versus* 10.8 months [6]. Hence, it is crucial to detect local recurrences early through follow-up measures. Studies have demonstrated that patients diagnosed with asymptomatic PDAC recurrence on surveillance computed tomography (CT) had better survival outcomes compared to symptomatic patients with recurrence [7, 8].

The primary challenge in detecting PDAC local tumor recurrence (LTR) on surveillance CT is to differentiate it from postoperative changes (POC). This presents a diagnostic challenge for radiologists as LTR and POC appear morphologically very similar as postoperative soft-tissue lesions (PSLs) [9]. Often, differentiation is only possible by observing progression of PSL on follow-up CT [9] or by performing an 18F-fluorodeoxyglucose positron emission tomography (PET)-CT, which has a higher sensitivity and specificity for LTR compared to contrast-enhanced CT [10]. Dual-energy (DE)-CT perfusion has only shown a non-significant lower trend of perfusion values of LTR compared to POC [11]. Furthermore, there is a significant correlation between perfusion CT parameters and iodine concentration (IC) in PDAC [12]. One study has investigated the potential of dual-energy computed tomography (DECT) to differentiate LTR from POC based on IC and CT numbers (HU) with promising results [13].

Not long ago, a photon-counting detector-CT (PCD-CT) system was approved for clinical use [14]. In a PCD, direct conversion occurs from x-ray photon to electrical signal, where ideally, each photon generates a separate signal. In a conventional energy-integrating detector (EID), however, indirect conversion occurs where the resulting electrical signal is an integrated signal generated from the energy deposition of all incident x-ray photons. Since the signal is proportional to the energy that is deposited by the incident photon, high-energy photons dominate the EID signal [15]. Due to the photoelectric effect, high-Z materials like iodine will exhibit a relatively high difference in attenuation from high to low photon energies compared to low-Z materials, such as soft tissues, which have a relatively energy-independent attenuation [16]. Consequently, contrast information carried by low-energy photons will be lost in EIDs. Since PCDs equally count low- and high-energy photons, a PCD-CT will reach a higher contrast for high-Z materials compared to an EID-CT [17]. Furthermore, PCDs provide energy information for every counted x-ray photon. By setting the energy threshold for photon counts above that of electronic noise, significant noise reduction can be achieved [15].

Early clinical experience of the inherent spectral capabilities of PCD-CT has demonstrated clinical benefits from iodine maps and virtual non-contrast images for quantification of myocardial extracellular volume, adrenal adenoma assessment, quantification of emphysema, and anemia detection [18]. Apart from certain technical limitations of PCDs related to count rate and dead time [19], the improved contrast and noise properties of PCDs could theoretically provide more accurate spectral capabilities compared to EID DECT systems, for instance, more reliable IC measurement. Phantom studies have demonstrated that IC measurement with PCD-CT is more accurate compared to DECT [20, 21]. To our knowledge, no previous study has investigated if PCD-CT-derived spectral data can differentiate between PDAC LTR and POC, as has been demonstrated with DECT [13]. Hence, the primary objective of this study was to determine if PCD-CT spectral variables, particularly IC and attenuation values on virtual monoenergetic images at different keV levels, could help discriminate between LTR and POC after PDAC resection. The secondary aim was to evaluate if a combination of different PCD-CT spectral variables could increase the diagnostic performance for predicting LTR.

## Methods

This retrospective study has received approval from the Swedish ethical review authority (Dnr 2023-01724-01, 2023-06715-02), and informed consent was waived owing to the retrospective nature of the study design.

### Patients

At our tertiary referral center at Karolinska University Hospital, Stockholm, Sweden, during the period 1 July 2022 to 31 October 2023, we retrospectively included 133 consecutive PCD-CT scans with PSLs around the peripancreatic vessels or at the operative site after resection of PDAC. Thirty-eight PCD-CT scans were excluded because they lacked spectral imaging data format (Spectral Post-Processing data). Sixteen PCD-CT scans were excluded because they were repeated on the same patient. Thus, 79 consecutive PCD-CT scans (79 patients) with PSL were included.

**Fig. 1:**
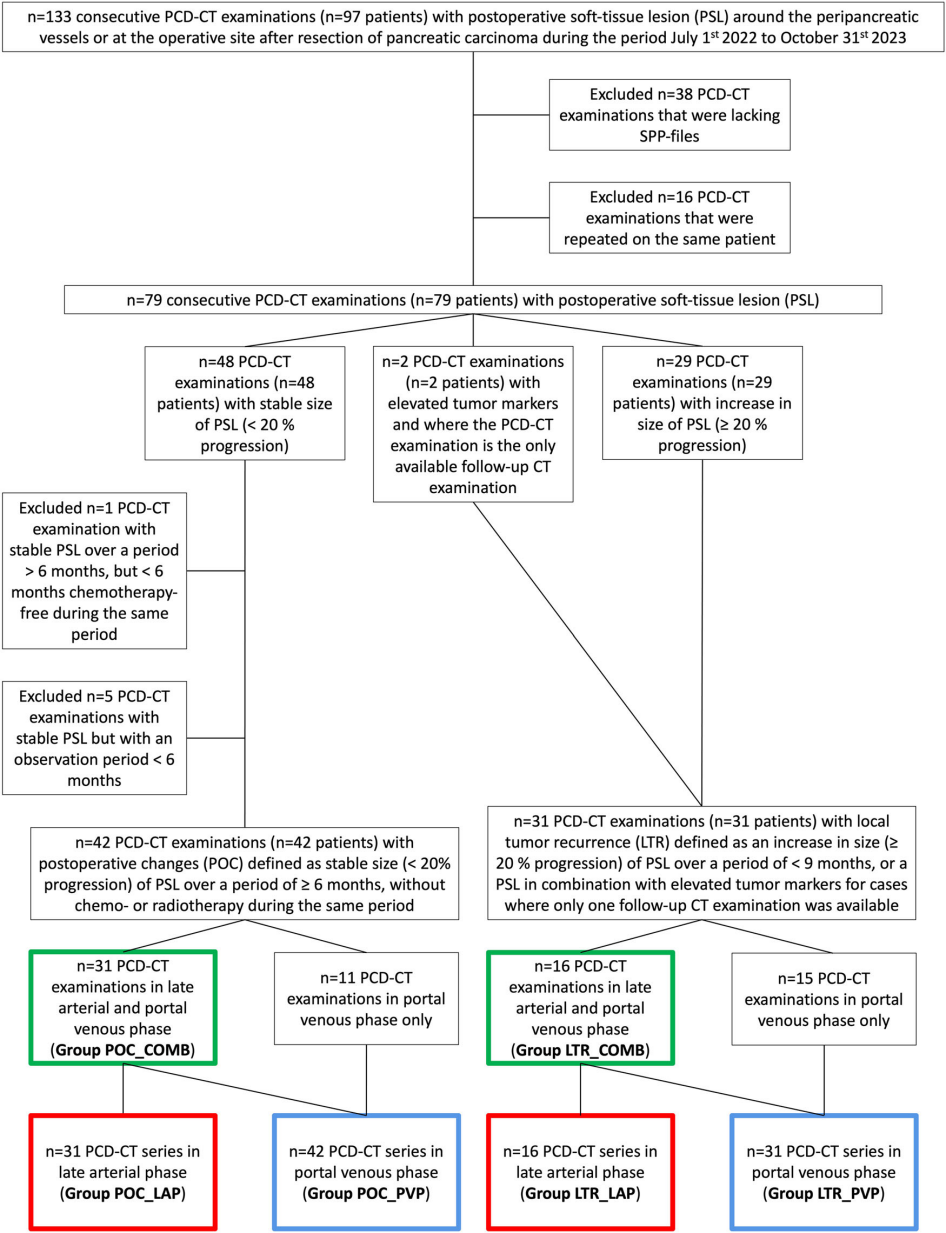
Flowchart of patient inclusion. Red framed boxes indicate groups in LAP (group POC_LAP/group LTR_LAP), blue framed boxes indicate groups in PVP (group POC_PVP/group LTR_PVP), and green framed boxes indicate groups in dual phase (group POC_COMB/group LTR_COMB). Each group pair was used to construct binary regression models predicting local tumor recurrence in the late arterial phase, portal venous phase, and dual phase, respectively. LAP, Late arterial phase; LTR, Local tumor recurrence; PCD-CT, Photon-counting detector computed tomography; POC, Postoperative changes; PSL, Postoperative soft-tissue lesion; PVP, Portal venous phase; SPP-files, Spectral post-processing files

In congruence with a prior DECT study, LTR was diagnosed by observing a ≥ 20% increase in the size of PSL along at least one dimension within a timeframe of < 9 months before and/or after the PCD-CT scan (*n* = 29) [13]. Alternatively, in instances where the PCD-CT scan was the only available surveillance CT scan, LTR was diagnosed by the presence of PSL in combination with elevated tumor marker (CA 19-9 > 37 kU/L) (*n* = 2). POC was diagnosed if the PSL was stable (< 20% progression in all dimensions) over a timeframe of ≥ 6 months before and/or after the PCD-CT scan, without concurrent administration of chemotherapy or radiotherapy. One PCD-CT scan was excluded because the patient was chemotherapy-free < 6 months, and five PCD-CT scans were excluded because the observation period was < 6 months. Finally, we included 31 PCD-CT scans with LTR and 42 with POC. Among the 31 PCD-CT scans with LTR, 16 were dual-phase scans, and 15 were in portal venous phase (PVP) only. The 16 late arterial phase (LAP) series from the dual-phase scans formed a group with LTR in LAP. The 16 PVP series from the dual-phase scans were combined with the 15 PVP-only series to form a group with LTR in PVP. Of the 42 PCD-CT scans with POC, 31 were dual-phase scans, and 11 were in PVP only. The 31 LAP series from the dual-phase scans formed a group with POC in LAP. The 31 PVP series from the dual-phase scans were combined with the 11 PVP-only series to form a group with POC in PVP. Binary regression models predicting LTR in LAP were constructed using variables from the LAP groups. Models for PVP utilized variables from the PVP groups. Additionally, combined models incorporating both LAP and PVP data were constructed using variables from the dual-phase scans. The patient inclusion process is illustrated in Fig. 1. An abdominal radiologist with 20 years’ experience in pancreatic imaging verified the diagnoses of LTR and POC, respectively.

### **PCD-CT protocol**

The scans were obtained utilizing a dual-source PCD-CT (NAEOTOM Alpha, Siemens Healthineers, Forchheim, Germany) with either a single PVP acquisition (70 s post contrast injection), or with a biphasic acquisition comprising a LAP (35–40 s post contrast injection) and then a PVP. The contrast medium iodixanol (Visipaque® 320 mgI/mL, GE Healthcare, Princeton, NJ, USA) was intravenously administered at a dose of 0.5 g of iodine per kg of body weight with a fixed injection time of 25 s. The maximum dosage weight was 100 kg for men and 80 kg for women.

We implemented the following scanning parameters: multi-energy scan mode (QuantumPlus, Siemens Healthineers); tube potential, 120 or 140 kV; automatic exposure control (CARE Dose4D, Siemens Healthineers); pitch, 0.8; rotation time, 0.5 s; collimation, 144 × 0.4 mm; kernel, Qr36 or Qr44; iterative reconstruction algorithm, quantum iterative reconstruction level 3. The CT series were reconstructed with a slice thickness of 0.6 mm.

### Post-processing of spectral data and quantitative analysis

**Fig. 2:**
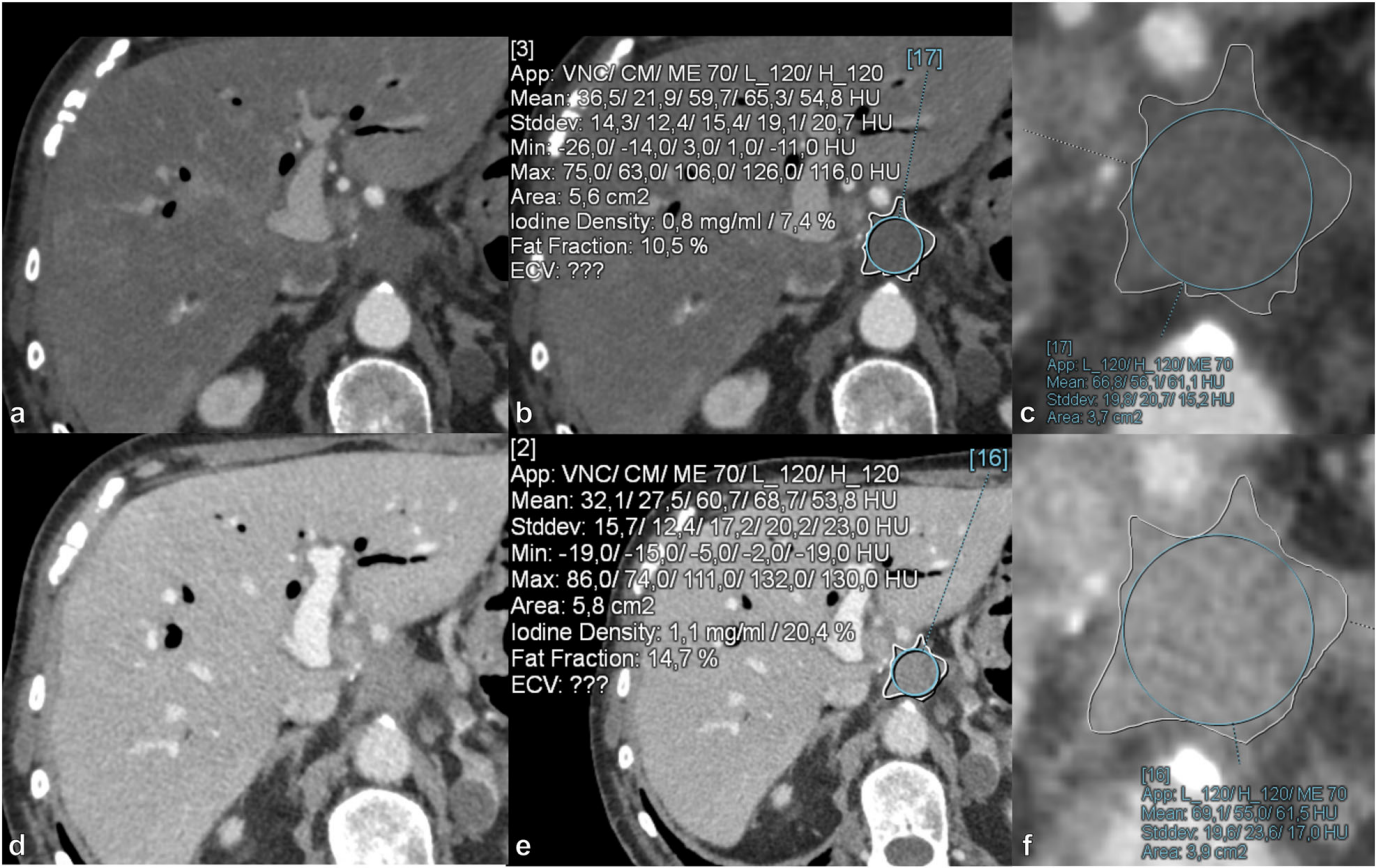
Axial contrast-enhanced abdominal PCD-CT scan in LAP in the first row (**a**, **b**, **c**), and in PVP in the second row (**d**, **e**, **f**). In the right column (**c**, **f**) are zoomed-in images of the ROIs. The images are of a 77-year-old male with LTR of PDAC post total pancreatectomy. Mean NIC in LAP was 0.08, and mean attenuation at 70 keV was 60.64 HU. According to the LAP model this yields a probability for LTR of 0.812 (81.2%). Mean IC in PVP was 1.03 mg/mL, and mean attenuation at 90 keV was 52.73 HU. According to the PVP model this yields a probability for LTR of 0.700 (70.0%). IC, Iodine concentration; LAP, Late arterial phase; LTR, Local tumor recurrence; NIC, Normalized iodine concentration; PCD-CT, Photon-counting detector computed tomography; PDAC, Pancreatic ductal adenocarcinoma; PVP, Portal venous phase; ROIs, Regions of interest

**Fig. 3:**
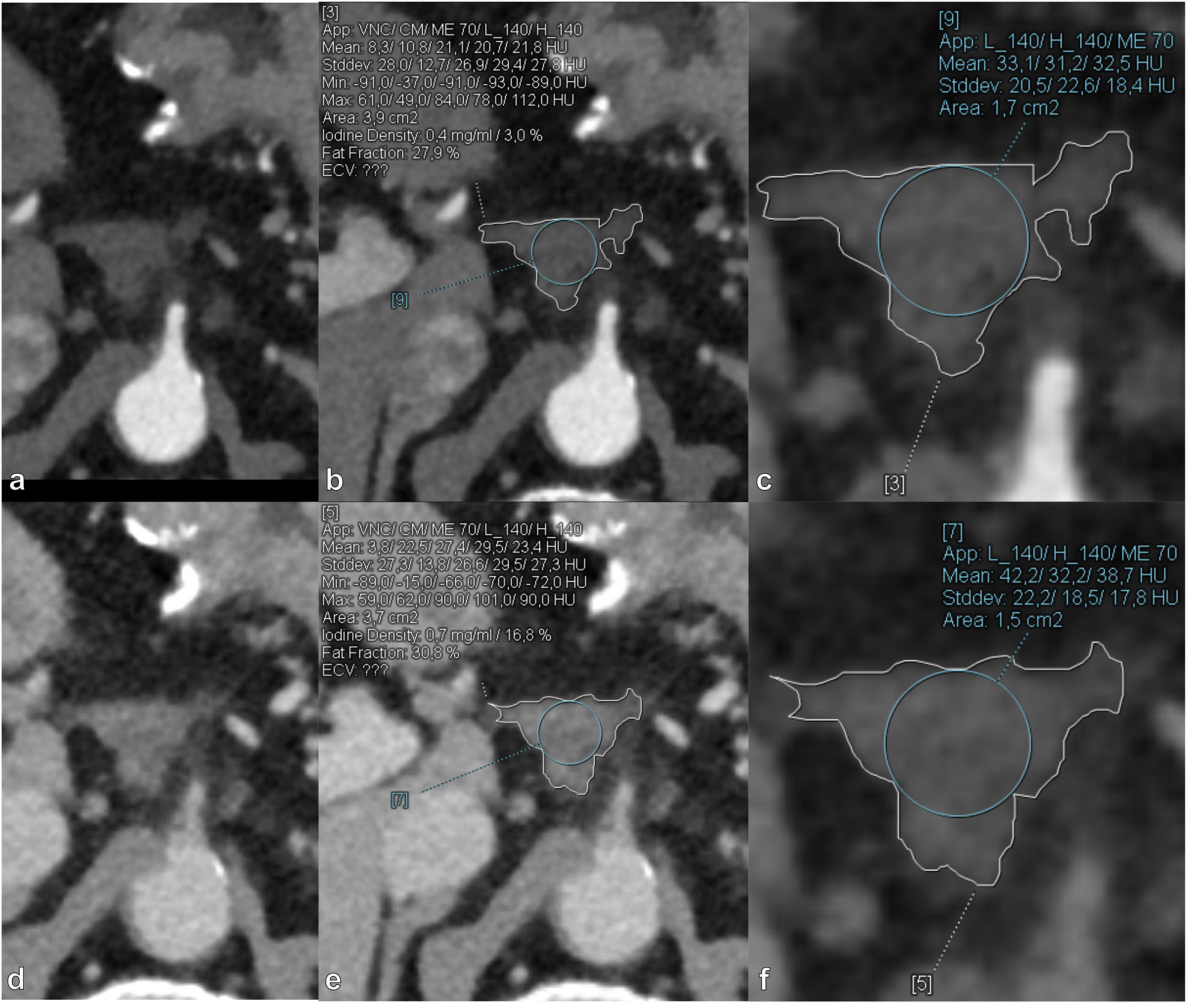
Axial contrast-enhanced abdominal PCD-CT scan in LAP in the first row (**a**, **b**, **c**), and in PVP in the second row (**d**, **e**, **f**). In the right column (**c**, **f**) are zoomed-in images of the ROIs. The images are of a 72-year-old male with POC post total pancreatectomy. Mean NIC in LAP was 0.03, and mean attenuation at 70 keV was 35.91 HU. According to the LAP model this yields a probability for LTR of 0.025 (2.5%). Mean IC in PVP was 0.80 mg/mL, and mean attenuation at 90 keV was 35.70 HU. According to the PVP model this yields a probability for LTR of 0.170 (17.0%). IC, Iodine concentration; LAP, Late arterial phase; LTR, Local tumor recurrence; NIC, Normalized iodine concentration; PCD-CT, Photon-counting detector computed tomography; POC, Postoperative changes; PVP, Portal venous phase; ROIs Regions of interest

Post-processing of spectral data, encompassing material decomposition and quantification of IC, fat fraction, and calculation of the slopes of HU curves, was conducted utilizing an imaging software (syngo.via, version VB80B, Siemens Healthineers). Within this software, the “Liver Virtual Non-Contrast” application was utilized to measure both the IC (mg/mL) and fat fraction (%). This application employs a modified three-material decomposition algorithm, with the base materials comprising liver tissue, fat, and iodine [22]. One radiologist with 10 years’ experience in abdominal imaging manually drew freehand regions of interest (ROIs) in each LTR and POC on five consecutive slices. The ROI was drawn as large as possible without extending beyond the confines of the lesion, while ensuring that vessels, calcifications, surgical clips, and artifacts, were excluded from the ROI (Figs. 2 and 3). For biphasic PCD-CT scans, the ROIs were placed in the identical location. From these ROIs the IC (mg/mL) and the fat fraction (%) were obtained. To mitigate any potential inter-patient variability stemming from injection rate, contrast agent dosage, and cardiac output, the IC was normalized relative to the IC within the abdominal aorta at the level of the superior mesenteric artery. This normalization was achieved by placing a circular ROI of maximal size within the aortic lumen, while carefully avoiding wall plaques. The normalized IC (NIC) was calculated by the formula: NIC = IC of lesion/IC of abdominal aorta.

In the syngo.via application “Monoenergetic Plus” only circular ROIs were available for HU-measurements. An as large as possible circular ROI was drawn within each abovementioned freehand ROI without exceeding the size of the latter. HU-values were obtained for virtual monoenergetic images ranging from 40 to 190 keV. We defined the slope of the spectral HU curve (*λ*_HU_) as the difference between the mean HU-value at 40 keV and the mean HU-value at 90 keV divided by the difference in energy (50 keV), according to the following formula:

$${{{\lambda }}}_{{{{\rm{HU}}}}}=\frac{{{{\rm{mean}}}} \, {{{{\rm{HU}}}}}_{40\,{{{\rm{keV}}}}}-{{{\rm{mean}}}} \, {{{{\rm{HU}}}}}_{90\,{{{\rm{keV}}}}}\,}{90\,{{{\rm{keV}}}}-40\,{{{\rm{keV}}}}}$$

We chose the range of 40 to 90 keV because the curve above 90 keV was nearly flat. Hence, the range from 40 kV to 90 keV offered a steeper slope with increased sensitivity to changes in attenuation.

The measurements obtained from the ROIs on the five consecutive slices were averaged by applying a 20% trimmed mean where the minimal and maximal values of the five measurements were excluded, and the remaining three values were averaged [23, 24].

The dimensions of LTR and POC were assessed by measuring their size in three perpendicular axes on the CT series in PVP, and the greatest percentage size difference along any axis was calculated.

Accurate positioning of ROIs and measurements was validated by an abdominal radiologist with 20 years’ experience.

### Statistical analysis

The statistical software IBM SPSS (v.28, Chicago, IL, USA) and R Studio (v. 2024.04.1+748, Posit Software, PBC) with the package “coin” (v. 1.4.3), “glmnet” (v. 4.1.8) and pROC (v. 1.18.5) were used to perform the data analysis. A stepwise approach was used to develop the prediction model for each contrast phase, as well as a combined model integrating variables from both phases. First, univariate analysis of seven PCD-CT spectral variables in LAP and PVP was performed comprising: IC, NIC, fat fraction, 40 keV (attenuation at 40 keV), 70 keV (attenuation at 70 keV), 90 keV (attenuation at 90 keV), and *λ*_HU_. The difference in each variable was compared between the LTR and POC groups. Levene’s test was used to evaluate the equality of variances (*i.e*., homoscedasticity) of the data in the LTR and POC groups, while the Shapiro-Wilk test was used to test for normality. Data exhibiting homoscedasticity and normality was compared between the groups using the independent samples *t*-test. Heteroscedastic normal data was compared between the groups with the Welch test (unequal variance *t*-test). Non-normal data was compared between the groups using a permutation-based two-sample test, implemented via the ‘independence_test()’ function from the “coin” package in R, with 10^8^ resampling iterations (‘nresample = 1e+08’) to approximate the null distribution [25, 26]. A two-sided significance level of 0.05 was set, and the *p*-values were Bonferroni-corrected. The continuous variables were presented as mean ± standard deviation. Second, all variables, including those not showing significant differences in the univariate analysis, were included in the least absolute shrinkage and selection operator (LASSO) regression analysis with ten-fold cross-validation to identify the most relevant predictors. Variables that were statistically significant in the univariate analysis and retained non-zero coefficients after LASSO regression were selected for further logistic regression analysis. Based on these methods, several binary logistic regression models were proposed. The Corrected Akaike Information Criterion score was utilized to identify the best-fitting models ensuring minimization of overfitting. Multicollinearity among predictor variables was checked by calculating the variance inflation factor. Predictor variables demonstrating a variance inflation factor > 10 were considered to exhibit problematic collinearity, and models containing such variables were excluded. Models that included predictor variables or an intercept that were not statistically significant (*p* > 0.05) were excluded. The linearity of the logit assumption for each model was tested by plotting the logit of the predicted probabilities against each predictor variable and visually assessing the relationship (Supplementary Fig. S1). The Hosmer-Lemeshow test was employed to evaluate the goodness-of-fit for the logistic regression models. A *p*-value > 0.05 was considered indicative of a good model fit.

Receiver operating characteristic (ROC) curve analysis was performed on the predictive models utilizing predicted probability values. Additionally, ROC curve analysis was conducted on variables that demonstrated statistical significance in the univariate analysis. The Youden index was calculated to suggest an optimal cutoff value that optimizes the balance between sensitivity and specificity for LTR detection [27]. Areas under the ROC curves (AUCs) were calculated, and the difference between the final models’ AUC and that of each PCD-CT spectral variable was evaluated with the DeLong test.

## Results

### Patients’ characteristics

The demographic characteristics of the patient population are depicted in Table 1. Thirty-one patients were diagnosed with LTR, 29 of which were identified based on the size progression of PSL according to the abovementioned criteria. For LTR cases, the mean observation period during which the progression of PSL was detected was 4.0 months, and the mean greatest size increase of PSL along any axis was 53.3%. In two patients, where the PCD-CT scan was the only available surveillance CT scan, tumor recurrence was identified based on the presence of a PSL combined with elevated tumor marker (one of the patients had a CA 19-9 of 1356 kU/L, and the other had a CA 19-9 of 376 kU/L). For cases with POC, the mean longest observation period without chemo- or radiotherapy during which the PSL was stable (< 20% size increase) was 23.6 months. Thirty POC cases demonstrated a decrease in the size of the PSL with a mean greatest size decrease along any axis of 17.0%. The mean longest lesion diameter at the start of the observation period was 31.3 mm for LTR and 27.3 mm for POC, without significant difference (*p* = 0.117). At the end of the observation period, the mean longest diameter was 39.8 mm for LTR and 25.8 mm for POC, a difference that was statistically significant (*p* < 0.001). The mean ROI areas were larger for LTR than POC, a finding that was statistically significant for all ROI areas except for freehand ROIs in LAP (freehand ROI LAP: 2.00 cm^2^ *versus* 1.43 cm^2^, *p* = 0.078; circular ROI LAP: 0.91 cm^2^ *versus* 0.51 cm^2^, *p* < 0.001; freehand ROI PVP: 2.27 cm^2^ *versus* 1.47 cm^2^, *p* = 0.031; circular ROI PVP: 0.96 cm^2^ *versus* 0.53 cm^2^, *p* = 0.031). There was a lower proportion of microscopically negative resection margins (R0) in LTR cases (12.9%) compared to POC cases (38.1%). Ten LTR cases and three POC cases had ongoing chemotherapy at the time of the PCD-CT. None of the LTR cases underwent biopsy to verify the LTR histologically.

**Table 1:** Demographic characteristics of the study population, in the LAP and PVP groups

	LTR (*n* = 31)	POC (*n* = 42)
Age (years)	67.9 ± 11.8 (37.0–85.1)	71.8 ± 9.6 (43.0–87.0)
Sex (male/female)	12/19 (38.7%/61.3%)	25/17 (59.5%/40.5%)
Surgery type, frequency (Whipple/Distal pancreatectomy/Total pancreatectomy)	21/4/6 (67.7%/12.9%/19.4%)	25/11/6 (59.5%/26.2%/14.3%)
R status, frequency (R0/R1/R2/Rx)	4/23/0/4 (12.9%/74.2%/0%/12.9%)	16/26/0/0 (38.1%/61.9%/0%/0%)
Observation period during which ≥ 20% size increase of PSL was detected (months)^a^	4.0 ± 1.8 (1.9–8.5)	N/A
Longest observation period without chemo- or radiotherapy during which there was < 20% size increase of PSL (months)	N/A	23.6 ± 18.6 (6.3–73.4)
Longest lesion diameter at beginning of observation period (mm)	31.3 ± 15.3 (15–87)	27.3 ± 13.1 (12–65)
Longest lesion diameter at end of observation period (mm)	39.8 ± 16.4 (19–87)	25.8 ± 12.5 (12–65)
Greatest size increase along any axis during observation period (%)	53.3 ± 36.7 (20.8–163.6)	9.1 ± 3.8 (5.3–14.3); (4 POC cases with an increase in size along any axis)
Greatest size decrease along any axis during observation period (%)	-6.9^b^	-17.0 ± 9.2 (-37.5 to -2.63); (30 POC cases with a decrease in size along any axis)
Ongoing chemotherapy at the time of PCD-CT, frequency (Yes/No)	10/21 (32.3%/67.7%)	3/39 (7.1%/92.9%)

Continuous variables are given as mean ± standard deviation (range)

*LTR* Local tumor recurrence, *N/A* Not applicable, *POC* Postoperative changes, *PSL* Postoperative soft-tissue lesion, *R status* Resection status

^a^ In two cases, where the PCD-CT scan was the only available surveillance CT scan, LTR was diagnosed by the presence of PSL in combination with elevated tumor marker

^b^ Only one LTR case demonstrated a size decrease along one axis, however, the greatest size increase along another axis in that same case was 45.5%

### Univariate analysis and LASSO regression

**Fig. 4:**
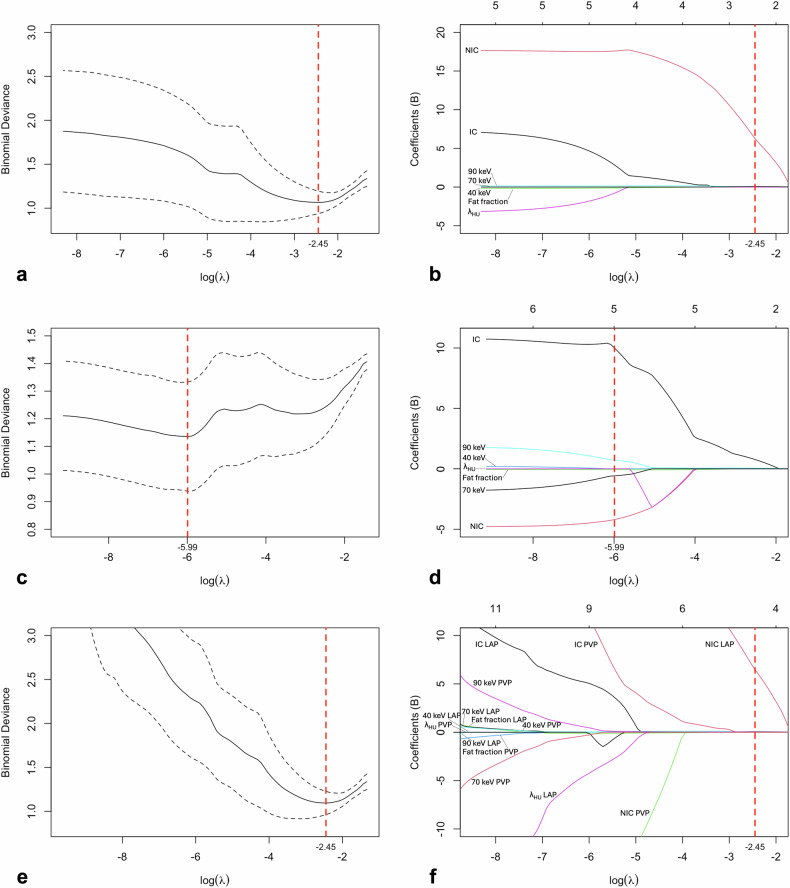
**a**, **c**, **e** Plots of LASSO regularization paths (**a** in LAP, **c** in PVP, and **e** in dual phase) showing the log(*λ*) associated with the minimum cross-validated error (deviance) as indicated by the vertical dashed red line. The continuous line represents the mean and dashed lines on either side of the continuous line indicate one standard error of the mean. **b**, **d**, **f** Plots of LASSO coefficient paths (**b** in LAP, **d** in PVP, and **f** in dual phase) where the individual coefficients are plotted as functions of log(*λ*). The vertical dashed red line indicates the log(*λ*) that minimizes model error. The variables that had non-zero coefficients at this log(*λ*) value and were statistically significant in the univariate analysis were included for further logistic regression analysis (NIC, 70 keV, and 90 keV in LAP; IC, 70 keV, and 90 keV in PVP; and NIC LAP, 70 keV LAP, and 90 keV PVP in dual phase). IC, Iodine concentration, LAP, Late arterial phase; LASSO, Least absolute shrinkage and selection operator; NIC, Normalized iodine concentration; PVP, Portal venous phase; *λ*_HU_, Slope of the spectral HU curve from 40 to 90 keV (mean HU40keV - mean HU90keV)/(90keV - 40 keV)

Of the seven variables in LAP, all but the variable fat fraction demonstrated significant differences between the LTR_LAP and POC_LAP groups. Of the seven variables in PVP, all but the variables NIC and fat fraction exhibited significant differences between the LTR_PVP and POC_PVP groups (Table 2). Of the 14 variables in the dual phase, seven were significantly different between LTR_COMB and POC_COMB (Supplementary Table S1). LASSO regression identified the optimal penalty parameter lambda (*λ*), which minimizes model error as log(*λ*) = -2.449 (*λ* = 0.08637233) for LAP and log(*λ*) = -5.991 (*λ* = 0.0025004) for PVP (Fig. 4). Statistically significant variables from the univariate analysis that retained non-zero coefficients after LASSO regression included three variables each for LAP (NIC, 70 keV, and 90 keV), PVP (IC, 70 keV, and 90 keV), and the dual phase (NIC in LAP, 70 keV in LAP, and 90 keV in PVP) (Fig. 4). Further logistic regression analysis, aimed at balancing goodness of fit and model complexity, resulted in the inclusion of the same selected variables in both the LAP model and the combined model. Hence, combining PCD-CT spectral variables from LAP and PVP did not improve the diagnostic performance of the model (Supplementary Table S2). The following two models offered the best diagnostic value for each contrast phase, respectively:

$${{P}} = 	 \,1/\left[1+{e}^{-(-10.479+18.830{{{\rm{X}}}}1+0.172{{{\rm{X}}}}2)}\right] \\ 	 \quad ({{{{\rm{LAP}}}}\; {{{\rm{model}}}};\; {{{\rm{X}}}}}1,{{{{\rm{NIC}}}};\; {{{\rm{X}}}}}2,\,70\,{{{\rm{keV}}}})$$

$${{P}} = 	 \,1/\left[1+{e}^{-(-7.478+2.595{{{\rm{X}}}}1+0.107{{{\rm{X}}}}2)}\right] \\ 	 \quad ({{{{\rm{PVP}}}}\; {{{\rm{model}}}};\; {{{\rm{X}}}}}1,{{{{\rm{IC}}}};\; {{{\rm{X}}}}}2,\,90\,{{{\rm{keV}}}})$$

**Table 2:** Comparison of quantitative spectral PCD-CT variables between LTR and POC in LAP and PVP

	LTR	POC	Uncorrected *p*-value	Bonferroni-corrected *p*-value
Late arterial phase				
IC (mg/mL)	0.90 ± 0.34	0.61 ± 0.25	0.001	0.009
NIC	0.12 ± 0.10	0.05 ± 0.03	< 0.001	0.002
Fat fraction (%)	17.14 ± 7.05	24.07 ± 8.47	0.007	ns (0.051)
Attenuation at 40 keV (HU)	94.72 ± 35.25	55.90 ± 23.54	< 0.001	0.004
Attenuation at 70 keV (HU)	55.87 ± 14.10	38.43 ± 13.01	< 0.001	< 0.001
Attenuation at 90 keV (HU)	47.47 ± 11.00	34.22 ± 12.03	< 0.001	0.004
*λ*_HU_ (HU/keV)	0.95 ± 0.57	0.43 ± 0.33	0.004	0.025
Portal venous phase				
IC (mg/mL)	1.12 ± 0.34	0.85 ± 0.25	< 0.001	0.002
NIC	0.23 ± 0.07	0.19 ± 0.07	ns (0.088)	ns (0.616)
Fat fraction (%)	18.53 ± 6.37	22.49 ± 7.56	0.021	ns (0.146)
Attenuation at 40 keV (HU)	108.97 ± 32.45	78.76 ± 21.34	< 0.001	< 0.001
Attenuation at 70 keV (HU)	59.36 ± 12.04	46.24 ± 11.13	< 0.001	< 0.001
Attenuation at 90 keV (HU)	49.20 ± 8.96	38.85 ± 9.84	< 0.001	< 0.001
*λ*_HU_ (HU/keV)	1.20 ± 0.54	0.80 ± 0.32	< 0.001	0.005

Variables are given as mean ± standard deviation

*IC* Iodine concentration, *LTR* Local tumor recurrence, *NIC* Normalized iodine concentration, *ns* Not significant, *POC* Postoperative changes, *λ*_HU_ Slope of the spectral HU curve from 40 to 90 keV (mean HU_40keV_ - mean HU_90keV_)/(90keV - 40 keV)

### ROC analysis

**Fig. 5:**
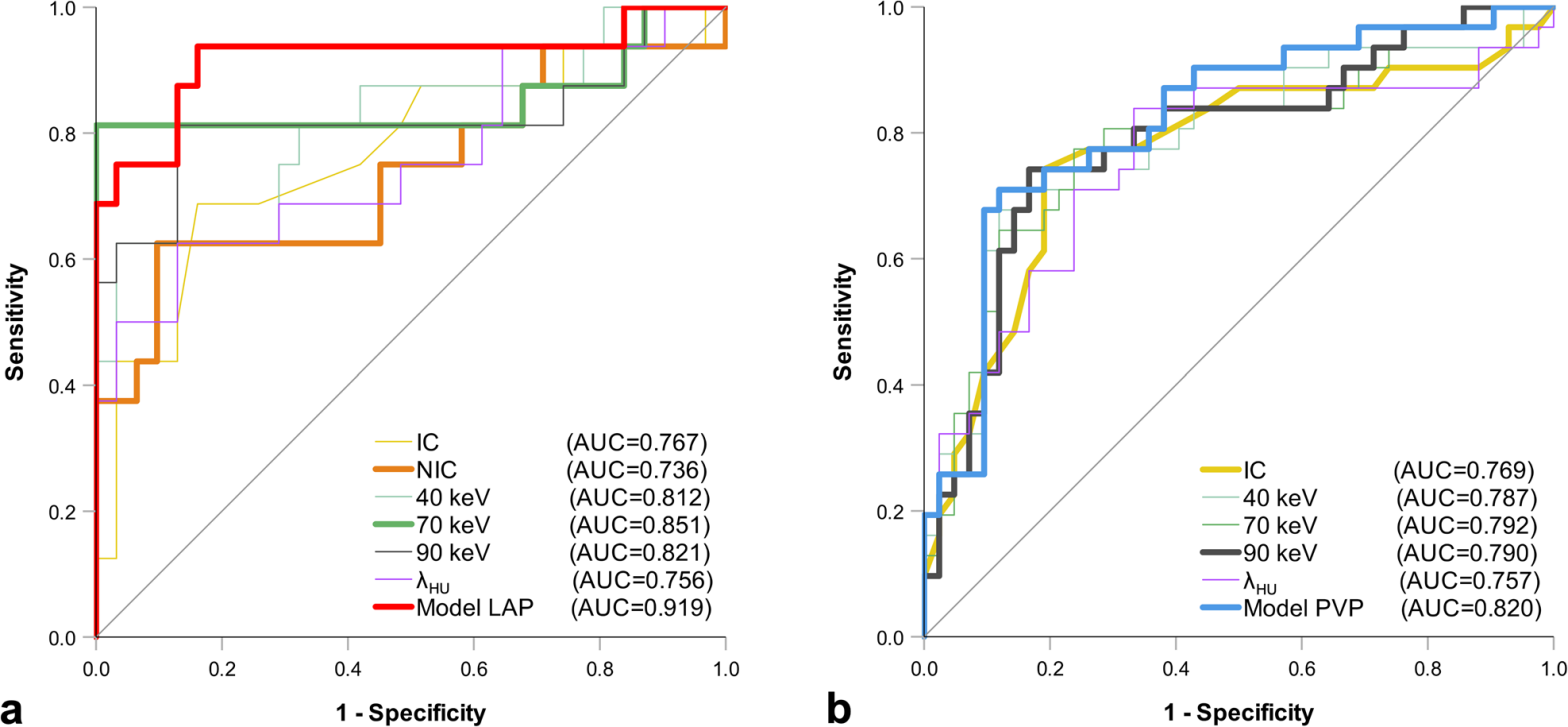
ROC analyses of spectral PCD-CT variables that were statistically significant in the univariate analysis and of the logistic regression models for LAP (**a**) and PVP (**b**), respectively, for predicting LTR post PDAC resection. Model LAP and Model PVP had the highest AUC values in comparison to any individual variable in LAP and PVP, respectively. AUC, Area under the curve; IC, Iodine concentration; LAP*,* Late arterial phase; LTR, Local tumor recurrence; NIC, Normalized iodine concentration; PCD-CT, Photon-counting detector computed tomography; PDAC, Pancreatic ductal adenocarcinoma; PVP, Portal venous phase; *λ*_HU_, Slope of the spectral HU curve from 40 to 90 keV (mean HU40keV - mean HU90keV)/(90keV - 40 keV); ROC, Receiver operating characteristic

The ROC analysis revealed that the AUC for the LAP model was 0.919 (95% CI 0.815–1.000), while for the PVP model, it was 0.820 (95% CI 0.720–0.921) (Fig. 5 and Table 3). The AUCs of the models were compared with those of the variables that showed statistical significance in the univariate analysis. In LAP, the AUC of the model was significantly higher compared to the AUCs of IC (*p* = 0.034), NIC (*p* = 0.013), and *λ*_HU_ (*p* = 0.018). No significant differences were observed in LAP between the AUCs of the variables NIC and 70 keV (*p* = 0.365), which served as predictors in the LAP model. In PVP, there were no significant differences between the AUC of the model and the AUCs of the individual variables.

**Table 3:** Performance of individual quantitative parameters and binary logistic regression models derived from PCD-CT spectral data for the differentiation between LTR and POC

	Differentiation between LTR and POC					
	AUC (95% CI)	*p*-value^a^	Cutoff value^b^	Sensitivity	Specificity	Accuracy
Late arterial phase						
IC (mg/mL)	0.767 (0.612–0.923)	**0.034**	0.800	68.8%	83.9%	78.7%
NIC	0.736 (0.567–0.904)	**0.013**	0.080	62.5%	90.3%	80.9%
Attenuation at 40 keV (HU)	0.812 (0.670–0.955)	0.053	92.170	62.5%	96.8%	85.1%
Attenuation at 70 keV (HU)	0.851 (0.695–1.000)	0.341	53.813	81.3%	100.0%	93.6%
Attenuation at 90 keV (HU)	0.821 (0.663–0.978)	0.188	44.515	81.3%	87.1%	85.1%
*λ*_HU_ (HU/keV)	0.756 (0.596–0.916)	**0.018**	0.799	62.5%	87.1%	78.7%
Model LAP	0.919 (0.815–1.000)	N/A	0.281	93.8%	83.9%	87.2%
Portal venous phase						
IC (mg/mL)	0.769 (0.651–0.886)	0.291	0.983	74.2%	81.0%	78.1%
Attenuation at 40 keV (HU)	0.787 (0.676–0.899)	0.150	103.318	67.7%	88.1%	79.5%
Attenuation at 70 keV (HU)	0.792 (0.683–0.901)	0.257	52.342	77.4%	76.2%	76.7%
Attenuation at 90 keV (HU)	0.790 (0.680–0.899)	0.401	47.818	74.2%	83.3%	79.5%
*λ*_HU_ (HU/keV)	0.757 (0.637–0.877)	0.110	0.862	83.9%	66.7%	74.0%
Model PVP	0.820 (0.720–0.921)	N/A	0.494	71.0%	88.1%	80.8%

*AUC* Area under the curve, *CI* Confidence interval, *IC* Iodine concentration, *LTR* Local tumor recurrence, *N/A* not applicable, *NIC* Normalized iodine concentration, *ns* Not significant, *PCD-CT* Photon-counting detector computed tomography, *POC* Postoperative changes, *λ*_HU_ Slope of the spectral HU curve from 40 to 90 keV (mean HU_40keV_ - mean HU_90keV_)/(90keV - 40 keV)

^a^ Comparison performed with the AUC of the LAP model and the PVP model, respectively, with the DeLong test. Bold type indicates statistical significance

^b^ Cutoff value determined using the Youden index

For the LAP model, at a cutoff predicted probability of 0.281, the sensitivity, specificity, and accuracy were 93.8%, 83.9%, and 87.2%, respectively. For the PVP model, at a cutoff predicted probability of 0.494, the sensitivity, specificity, and accuracy were 71.0%, 88.1%, and 80.8%, respectively (Fig. 5 and Table 3).

## Discussion

In our study, PCD-CT-derived spectral data has been used for the first time to discriminate between LTR and POC following resection of PDAC. LTR demonstrated significantly higher mean IC in both contrast phases and significantly higher mean NIC in LAP. In LAP, LTR cases exhibited more than double the mean NIC of POC cases. This finding is in line with a previous DECT study that demonstrated significantly higher iodine uptake in LTR compared to POC in “early venous phase” (images acquired 30 s after trigger time point) [13], which corresponds to our LAP (35–40 s after contrast injection).

As far as we know, this study represents the first evaluation of spectral CT variables in a later contrast phase (*i.e*., PVP, 70 s after contrast injection) for discriminating between PDAC LTR and POC. However, our findings reveal that the diagnostic performance of spectral variables in PVP is comparatively modest when compared to the corresponding variables in LAP. Furthermore, while significant differences in mean IC were evident between LTR and POC during the venous phase, normalization of these values (NIC) weakened the statistical significance, unlike the LAP, where normalization increased statistical significance. The LAP is an earlier phase and, therefore, more sensitive than the PVP to variations in cardiac output and blood pressure.

The observed increase in statistical significance of differences after normalization of IC in LAP indicates that LTR truly has a higher contrast uptake than POC during this phase. Conversely, the non-significant difference in NIC between LTR and POC in PVP suggests that the significant difference in absolute IC values may be attributed to variability in systemic patient factors. Nevertheless, IC in PVP was significantly different between LTR and PVP and was also selected as a predictor variable by the LASSO regression analysis.

Furthermore, the attenuation values were significantly higher for LTR compared to POC in both contrast phases, with the greatest attenuation difference between LTR and POC observed in LAP. This observation, coupled with the significant difference in NIC in LAP, could be due to the already established fact that the contrast enhancement in PDAC increases most during 0–30 s (corresponding to the LAP) and then plateaus after 60 s (corresponding to the PVP) [28].

Given the previously established significant correlation between perfusion CT parameters (blood volume and permeability) and IC in PDAC [12], our findings suggest that vascularization is higher in LTR compared to POC, as also proposed in the previous DECT study [13]. This contrasts with the findings from the initial DECT perfusion study that presented lower perfusion values of LTR compared to POC, indicating a poorer vascular supply of LTR [11], and this discrepancy was also pointed out by the authors of the previous DECT study [13]. The reason for these contradicting results is unclear. Possible sources of measurement error when assessing relatively small lesions near larger vessels on an abdominal CT perfusion scan are breathing artifacts, even though the authors tried to mitigate these by instructing the patients to implement shallow breathing [11].

It is well established that PDAC has a low microvascular density compared to other malignant tumors. Furthermore, the pronounced fibro-inflammatory reaction (desmoplastic reaction) causes vessel collapse. This results in a hypoxic microenvironment that is profibrogenic, but that also triggers pancreatic stellate cells to produce angiogenic factors [29]. Less is known about the morphology and composition of intra-abdominal POC. To our knowledge, nothing has yet been published about the morphology of postoperative retroperitoneal changes. We have found three studies on peritoneal adhesions that challenge previous beliefs that adhesions are merely composed of avascular fibrous scar tissue. These studies demonstrated histologically that apart from containing collagen bundles, peritoneal adhesions are cellular and vascularized structures [30–32]. Histological comparison of microvascular density between PDAC and POC is a topic that requires further investigation.

We found that the fat fraction was lower for LTR compared to POC in both contrast phases; however, this difference was not statistically significant. This is in line with the previous DECT study [13].

We have demonstrated that integrating the two LAP variables, NIC and 70 keV, in the LAP model resulted in a higher AUC compared to the AUCs obtained from each variable independently. The increase was statistically significant for the variable NIC. In line with the previous DECT study, we did not observe any significant difference in the AUCs between the NIC and 70 keV variables in LAP. We chose to evaluate the 70 keV virtual monoenergetic images since they are equivalent to 120 kVp images [33]. Also, the mean energy of the photons at 120 kVp is around 70 keV [34].

While further validation is necessary, the LAP model, in its current form, demonstrates higher sensitivity and specificity compared to conventional contrast-enhanced CT without spectral information, which, according to a meta-analysis, showed a pooled sensitivity of 70% and specificity of 80%. Our LAP model also outperforms 18F-fluorodeoxyglucose PET-CT in terms of sensitivity, as reported in the same study where 18F-fluorodeoxyglucose PET-CT had a pooled sensitivity of 88% and specificity of 89% [10]. Compared to other 18F-fluorodeoxyglucose PET-CT studies, however, our model exhibited inferior diagnostic performance, with one study demonstrating a sensitivity of 97.6% and an accuracy of 90% [35], and another study reporting a sensitivity of 90.9%, a specificity of 100.0% and an accuracy of 92.3% [36].

There are several limitations of our study. First, we have a relatively small sample size. However, PDAC is a rare diagnosis, which, coupled with our strict inclusion criteria, further diminishes the pool of eligible patients. Second, none of the PSLs were biopsied. This is because we follow PSLs at our center with CT, and in cases of progression, we only consider biopsy for resectable lesions. None of the LTR cases in our cohort were considered for re-resection (25 cases had metastatic disease, 5 cases had no metastases but inoperable LTR, and 1 case received the best supportive care due to high age (85 years)). Third, 10 of 31 patients with LTR and 3 of 42 patients with POC had ongoing chemotherapy at the time of the PCD-CT scan. Despite this, we decided to include these patients, given the routine administration of adjuvant chemotherapy in most cases. Fourth, in some cases, PSLs were in the immediate vicinity of central abdominal vessels, introducing measurement uncertainty. We tried to mitigate this by making sure that the freehand ROIs are drawn within the confines of the PSL, excluding any vessels, and by applying a 20% trimmed mean of measurements from the five consecutive slices to minimize the influence of outliers. Fifth, the discrepancy in case numbers between the PVP groups (31 LTR cases, 42 POC cases) and LAP groups (16 LTR cases, 31 POC cases) hinders a direct comparison of diagnostic performance between the LAP model and the PVP model. However, given the rarity of this disease, we included the additional PVP-only cases to boost the statistical power of the PVP model, as this later contrast phase has not been assessed in this context in the literature. Furthermore, we created groups comprising patients scanned in both LAP and PVP (group LTR_COMB, *n* = 16; and group POC_COMB, *n* = 31), where univariate and logistic regression analysis yielded identical predictor variables as those in the LAP model (NIC in LAP, and 70 keV in LAP). Given the absence of PVP variables in the final model of the combined phase groups, we abstained from further direct comparisons between LAP and PVP variables.

Our study has several advantages over previous research. First, we performed quantitative measurements on CT series with a 0.6-mm slice thickness, unlike the previous DECT study [13], which used a 5-mm slice thickness, thus mitigating partial volume effects. Second, we normalized the IC of the PSL to the IC of the abdominal aorta to minimize inter-patient variations in physiology and contrast administration. The inclusion of NIC as a predictor variable in the LAP model likely increases the robustness and generalizability of this model. We chose not to normalize the attenuation values (variables 40 keV, 70 keV, and 90 keV) to those of the aorta, as it would introduce an additional layer of complexity to the models, and we are unaware of any studies that have calculated the slope of the spectral curve based on normalized HU-values. However, we are planning to perform the normalization of these variables as well in a forthcoming study. We also intend to include a larger number of patients who have undergone PCD-CT in both contrast phases in a future study to further validate the models.

In conclusion, quantitative variables derived from PCD-CT spectral data facilitate the discrimination of LTR from POC in LAP and PVP following PDAC resection. Combining relevant variables in a multivariable binary logistic regression model enhances diagnostic performance, particularly in LAP, where the model at the optimal cutoff value achieves a 94% sensitivity, an 84% specificity, and an 87% accuracy.

## Supplementary information

**Additional file 1: Supplementary Table S1.** Comparison of quantitative spectral PCD-CT variables between LTR and POC in dual phase. **Supplementary Table S2.** Comparison between different logistic regression models for late arterial phase, portal venous phase, and both contrast phases combined, comprising variables that were statistically significant in the univariate analysis and retained non-zero coefficients after LASSO regression. **Supplementary Fig. S1.** Check of the linearity of the logit assumption for the LAP model (**a**, **b**) by plotting the logit of predicted probabilities against the variable NIC (**a**) and 70 keV (**b**); and for the PVP model (**c**, **d**) by plotting the logit of predicted probabilities against the variable IC (**c**) and 90 keV (**d**). The variables from each model demonstrate a satisfactory level of linearity with the logit of predicted probabilities.

## Data Availability

The datasets used in this study are available from the corresponding author upon reasonable request.

## Abbreviations

AUC: Area under the curve
CT: Computed tomography
DECT: Dual-energy computed tomography
EID: Energy-integrating detector
IC: Iodine concentration
LAP: Late arterial phase
LASSO: Least absolute shrinkage and selection operator
LTR: Local tumor recurrence
NIC: Normalized iodine concentration
PCD: Photon-counting detector
PCD-CT: Photon-counting detector computed tomography
PDAC: Pancreatic ductal adenocarcinoma
PET-CT: Positron emission tomography-computed tomography
POC: Postoperative changes
PSL: Postoperative soft-tissue lesion
PVP: Portal venous phase
ROC: Receiver operating characteristic
ROI: Region of interest

## Declarations

### Study subjects or cohorts overlap

Eight study subjects are also included in another study cohort with a different objective.
