# Supplementary material for: Contrast-enhanced photon-counting detector CT for discriminating local recurrence from postoperative changes after resection of pancreatic ductal adenocarcinoma
Source: Eur Radiol Exp. 2025 Feb 22;9:26. doi: 10.1186/s41747-025-00567-0 (PMC11846822; doi:10.1186/s41747-025-00567-0)
Supplement: Supplementary file 1 — Additional file 1: Supplementary Table S1. Comparison of quantitative spectral PCD-CT variables between LTR and POC in dual phase. Supplementary Table S2. Comparison between different logistic regression models for late arterial phase, portal venous phase, and both contrast phases combined, comprising variables that were statistically significant in the univariate analysis and retained non-zero coefficients after LASSO regression. Supplementary Fig. S1. Check of the linearity of the logit assumption for the LAP model (a, b) by plotting the logit of predicted probabilities against the variable NIC (a) and 70 keV (b); and for the PVP model (c, d) by plotting the logit of predicted probabilities against the variable IC (c) and 90 keV (d). The variables from each model demonstrate a satisfactory level of linearity with the logit of predicted probabilities. [file 41747_2025_567_MOESM1_ESM.pdf]

Contrast-enhanced photon-counting detector CT for discriminating local recurrence from postoperative changes after resection of pancreatic ductal adenocarcinoma

ELECTRONIC SUPPLEMENTARY MATERIAL

Supplementary Table S1. Comparison of quantitative spectral PCD-CT variables between LTR and POC in dual phase

	LTR	POC	Uncorrected *p*-value	Bonferroni-corrected *p*-value
IC (mg/mL) in LAP	0.90 ± 0.34	0.61 ± 0.25	0.001	0.017
NIC in LAP	0.12 ± 0.10	0.05 ± 0.03	< 0.001	0.005
Fat fraction (%) in LAP	17.14 ± 7.05	24.07 ± 8.47	0.007	ns (0.102)
Attenuation at 40 keV (HU) in LAP	94.72 ± 35.25	55.90 ± 23.54	< 0.001	0.009
Attenuation at 70 keV (HU) in LAP	55.87 ± 14.10	38.43 ± 13.01	< 0.001	0.001
Attenuation at 90 keV (HU) in LAP	47.47 ± 11.00	34.22 ± 12.03	< 0.001	0.007
λ_{HU} (HU/keV) in LAP	0.95 ± 0.57	0.43 ± 0.33	0.004	ns (0.051)
IC (mg/mL) in PVP	1.12 ± 0.43	0.86 ± 0.26	0.034	ns (0.477)
NIC in PVP	0.24 ± 0.09	0.20 ± 0.08	ns (0.137)	ns (1)
Fat fraction (%) in PVP	17.38 ± 6.81	23.02 ± 8.14	0.022	ns (0.308)
Attenuation at 40 keV (HU) in PVP	112.23 ± 42.11	78.35 ± 21.25	0.007	ns (0.097)
Attenuation at 70 keV (HU) in PVP	60.82 ± 15.15	45.65 ± 11.17	< 0.001	0.004
Attenuation at 90 keV (HU) in PVP	50.27 ± 10.76	38.28 ± 10.02	< 0.001	0.006
λ_{HU} (HU/keV) in PVP	1.24 ± 0.70	0.80 ± 0.32	0.028	ns (0.394)

IC Iodine concentration, *LAP* Late arterial phase, *LTR* Local tumor recurrence, *NIC* Normalized iodine concentration, *ns* Not significant, *POC* Postoperative changes, *PVP* Portal venous phase, λ_{HU} Slope of the spectral HU curve from 40 to 90 keV (mean HU_{40keV} − mean HU_{90keV})/(90keV − 40keV)

Supplementary Table S2. Comparison between different logistic regression models for late arterial phase, portal venous phase, and both contrast phases combined, comprising variables that were statistically significant in the univariate analysis and retained non-zero coefficients after LASSO regression

	Is there multicollinearity?, (highest VIF)	AICc	Nagelkerke R^2	Highest *p*-value among predictor variables	*p*-value of intercept	ROC AUC	*p*-value of Hosmer-Lemeshow test
Late arterial phase							
NIC + 70 keV + 90 keV	Yes, (23.383)	37.510	0.679	0.861	0.003	0.944	< 0.001
NIC + 70 keV*	No, (1.004)	37.450	0.643	0.005	0.002	0.919	0.073
NIC + 90 keV	No, (1.001)	35.148	0.679	0.005	0.003	0.944	< 0.001
70 keV + 90 keV	Yes, (18.961)	49.064	0.436	0.591	0.003	0.843	0.019
NIC	N/A	52.168	0.321	0.010	0.001	0.736	0.467
70 keV	N/A	47.071	0.430	0.002	0.001	0.851	< 0.001
90 keV	N/A	49.861	0.372	0.003	0.002	0.821	0.006
Portal venous phase							
IC + 70 keV + 90 keV	Yes, (66.530)	69.917	0.548	0.004	< 0.001	0.889	0.395
IC + 70 keV	No, (1.296)	82.122	0.373	0.082	< 0.001	0.801	0.158
IC + 90 keV*	No, (1.078)	79.029	0.414	0.012	< 0.001	0.820	0.338
70 keV + 90 keV	Yes, (15.140)	85.416	0.329	0.855	< 0.001	0.797	0.462
IC	N/A	89.766	0.234	0.001	< 0.001	0.769	0.035
70 keV	N/A	83.272	0.328	< 0.001	< 0.001	0.792	0.445
90 keV	N/A	84.423	0.312	< 0.001	< 0.001	0.790	0.185
Late arterial and portal venous phase							
NIC LAP + 70 keV LAP + 90 keV PVP	No, (3.109)	38.475	0.665	0.259	0.002	0.925	0.085
NIC LAP + 70 keV LAP*	No, (1.004)	37.450	0.643	0.005	0.002	0.919	0.073
NIC LAP + 90 keV PVP	No, (1.002)	38.904	0.620	0.009	0.001	0.913	0.189
70 keV LAP + 90 keV PVP	No, (3.011)	49.171	0.434	0.667	0.001	0.851	< 0.001
NIC LAP	N/A	52.168	0.321	0.010	0.001	0.736	0.467
70 keV LAP	N/A	47.071	0.430	0.002	0.001	0.851	< 0.001
90 keV PVP	N/A	51.430	0.337	0.003	0.002	0.790	0.186

AICc Corrected Akaike Information Criterion, *AUC* Area under the curve, *IC* Iodine concentration, *LAP* Late arterial phase, *LASSO* Least absolute shrinkage and selection operator, *N/A* not applicable, *NIC* Normalized iodine concentration, *PVP* Portal venous phase, *VIF* Variance inflation factor

* Chosen model with the best balance between goodness of fit and model complexity

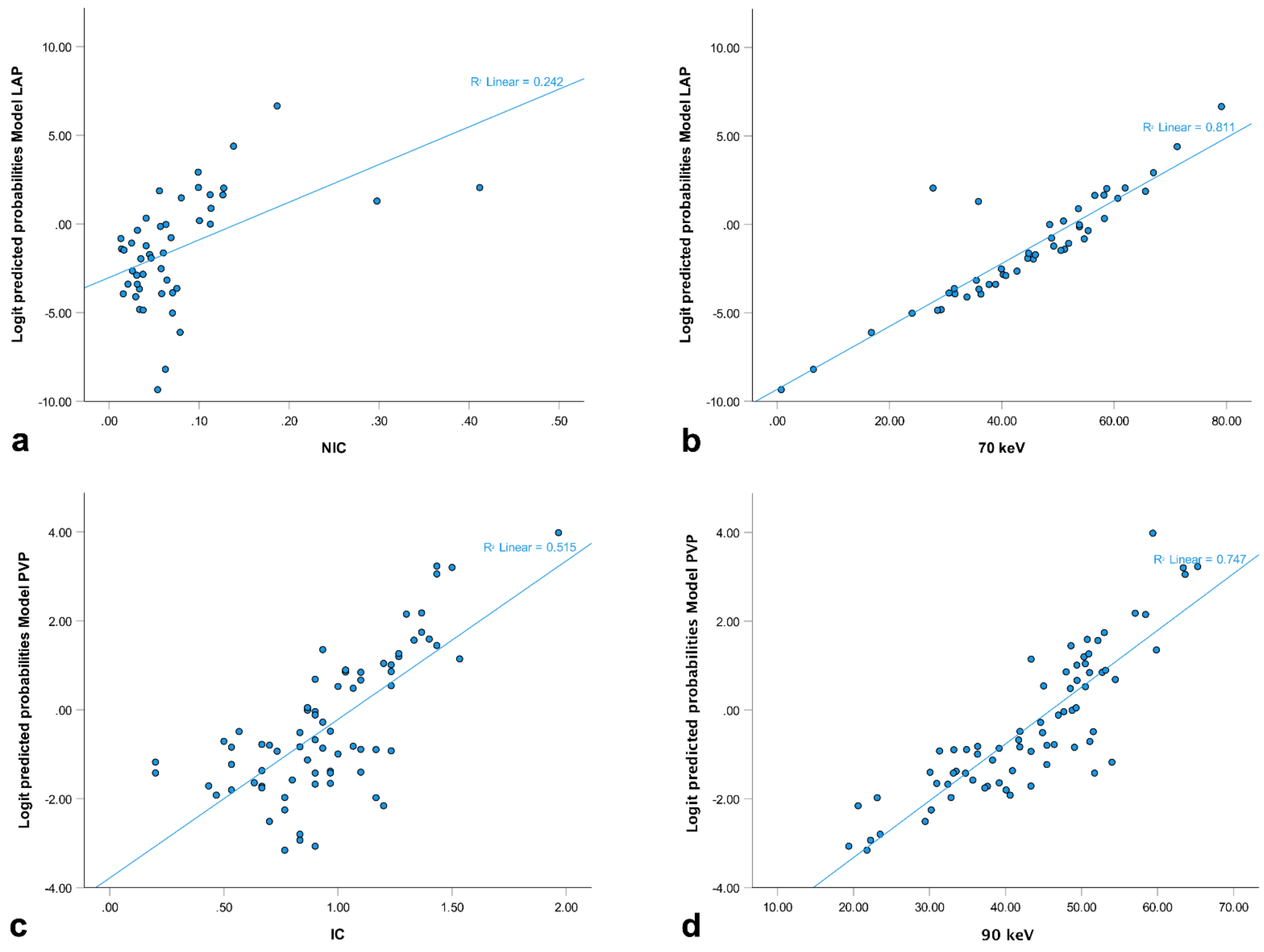

Supplementary Fig. S1. Check of the linearity of the logit assumption for the LAP model (**a, b**) by plotting the logit of predicted probabilities against the variable NIC (**a**) and 70 keV (**b**); and for the PVP model (**c, d**) by plotting the logit of predicted probabilities against the variable IC (**c**) and 90 keV (**d**). The variables from each model demonstrate a satisfactory level of linearity with the logit of predicted probabilities. *IC* Iodine concentration, *LAP* Late arterial phase, *NIC* Normalized iodine concentration, *PVP* Portal venous phase

Eur Radiol Exp (2025) Alagic Z, Valls Duran C, Svensson-Marcial A, Koskinen SK.
